# Choosing Wisely: A Systematic Review of Terlipressin Versus Octreotide for Variceal Bleeding

**DOI:** 10.7759/cureus.86973

**Published:** 2025-06-29

**Authors:** Shivling S Swami, Alousious Kasagga, Amanuel K Assefa, Maysaa N Amin, Rahma Hashish, Khaled Agha Tabari, Ann K Yu

**Affiliations:** 1 Internal Medicine, California Institute of Behavioral Neurosciences & Psychology, Fairfield, USA; 2 Pathology, Peking University, Beijing, CHN; 3 Orthopedics and Trauma, University Hospitals of Leicester NHS foundation trust, Leicester, GBR; 4 Microbiology/Immunology, California Institute of Behavioral Neurosciences & Psychology, Fairfield, USA; 5 Internal Medicine, Sherwood Forest NHS Trust, Nottingham, GBR; 6 Radiology, Queen Elizabeth University Hospital, Glasgow, GBR

**Keywords:** endoscopic banding, endoscopic ligation, octreotide, terlipressin, upper gi bleeding, variceal bleeding, vasoactive agents

## Abstract

High pressure in the portal circulation is a key indicator of advanced liver disease. It has many harmful effects on patients with cirrhosis, such as promoting the growth of dilated esophageal vessels. As portal pressure rises, it creates serious risks for life-threatening bleeding problems from variceal sources, which leads to higher mortality and worse patient outcomes. A multidisciplinary approach that includes pharmacologic and endoscopic procedures and radiological techniques is necessary to treat variceal hemorrhage effectively. There are typically three main components to clinical management: primary prophylaxis, which is the initial step in preventing disease; secondary prophylaxis, which is the second step; and acute hemorrhage management, which is the third step. The goal of both primary and secondary prophylactic measures is to stop variceal bleeding episodes from happening. Acute variceal hemorrhage is a severe medical condition that needs immediate treatment to stop the bleeding and prevent it from happening again. Vasoactive drugs are the primary treatment for all patients with suspected variceal bleeding. They are safe and simple to give, and they are part of standard treatment protocols. When bleeding continues during an endoscopic procedure, there is strong evidence that vasoactive drugs should be given right away, preferably before the diagnostic endoscopy. There is a lack of direct comparative studies in the current literature that look at the relative therapeutic benefits of vasoactive drugs when used as adjunctive therapies with endoscopic interventions for managing variceal bleeding. The goal of this systematic review was to look at and compare the safety and effectiveness of various vasoactive drugs as supportive treatments for patients with esophageal variceal hemorrhage who were also receiving endoscopic therapy. We used a search strategy that included several electronic databases, such as PubMed, PubMed Central, ScienceDirect, and Google Scholar, to find studies that investigated how well these two drugs worked in treating active variceal bleeding outcomes. The review included a range of study types, such as meta-analyses, observational studies, case-control studies, systematic reviews, narrative reviews, and randomized controlled trials (RCTs). A total of 22,422 participants were included in the final analysis of 14 studies, including two RCTs, two observational studies, seven meta-analyses, and three narrative reviews. The results revealed that various vasoactive drugs had similar therapeutic effects on controlling bleeding, length of hospital stay, death rates, and adverse event profiles when used as adjunctive therapies. The research demonstrated that vasoactive medication treatment results in improved control of bleeding and shorter hospital stays. It also led to lower rates of immediate death from all causes and fewer blood product requirements. Many studies comparing these drugs found that they all worked similarly in clinical settings. Further research is therefore needed, such as high-quality randomized controlled trials, to strengthen these conclusions.

## Introduction and background

Among patients with cirrhosis and elevated portal pressures, hemorrhage from dilated gastroesophageal veins represents the leading cause of mortality and constitutes a critical complication requiring appropriate, timely intervention. These patients may experience rapid clinical deterioration leading to fatal outcomes [1-4]. Data indicates that approximately 5-15% of patients with liver cirrhosis experience variceal bleeding each year, comprising roughly 70% of all upper digestive tract hemorrhagic incidents [2,3,5]. There is an occurrence of bleeding in around 50% of the patients who have cirrhosis at some point in the disease course [6]. Esophageal varices are prevalent in approximately 30% of patients with compensated cirrhosis and 60% in those with decompensated cirrhosis [7]. Within the first year of diagnosis, 12% of patients with varices experience esophageal variceal bleeding (EVB); this number increases to 33% after three years [8].

In the past, the mortality rate for untreated variceal hemorrhage ranged from 30% to 50% [9,10]. Currently, the outcomes have improved, and combination therapy (vasoactive medications and endotherapy) can control 75% of bleeding. Despite advancements in endoscopic procedures and drug therapies, advanced hepatic cirrhosis continues to demonstrate mortality rates of 15-20%, although these figures have shown a decline over recent decades. It is still greater than the death rate from myocardial infarction [2,3,7,9]. Around 50% of patients who survive the initial episode may have a repeat episode of bleeding within one year of the initial episode [10]. Improved survival rates in patients with upper gastrointestinal bleeding can be attributed to higher standards of medical care and prophylactic use of antibiotics, which has led to a reduction of infection by 32% and mortality of 9% [9]. Patients who experience rebleeding within five days or whose acute bleeding episodes cannot be initially controlled have a greater mortality rate [2]. Recurrent variceal bleeding occurs after 24 hours following cessation of bleeding in about 30-40% of cases within six weeks of the primary bleeding episode [6]. The objective of pharmacological intervention for acute esophageal variceal bleeding is to reduce splanchnic blood flow and lower portal vascular pressure.

Current treatment options for management of esophageal variceal hemorrhage include endoscopic procedures, trans jugular intrahepatic portosystemic shunts (TIPS), pharmacologic interventions (vasoactive drugs and nonselective beta-blockers), and shunt surgery. Variceal bleeding is always a medical emergency, and treatment should begin as soon as it is recognized, ideally at the patient's residence or while being transported to the hospital. For all patients with suspected variceal hemorrhage, therapy with a vasoactive medication is the primary line of treatment due to its ease of administration and safety. Vasoactive medications should be used alongside endoscopic therapy and continued for up to five days. This timeframe is very important, since nearly 25% of mortality occurs in the initial stages, particularly within five days, which is a period when early rebleeding is most common [6,11]. Since the bleeding in 26-56% of individuals will have a nonvariceal etiology, which includes pathologies like peptic ulcers and portal hypertensive gastropathy, endoscopy is necessary to make an appropriate diagnosis.

There are compelling reasons to believe that the optimal course of treatment is to begin vasoactive medication as soon as feasible, and then endoscopic therapy should follow, especially if there is active bleeding throughout the endoscopy. Pretreatment with vasoactive medication prior to endoscopy streamlines the procedure, enhances bleeding control, and reduces the risk of rebleeding within the first five days [12]. Despite a trend towards increased survival with combination therapy, survival at six weeks has not improved. The benefit of continuing vasoactive agents for five days following the initial bleed, particularly when no active bleeding is seen, remains uncertain [3]. Either increasing the resistance to variceal blood flow within the varices or decreasing variceal blood flow can reduce variceal pressure. Changes in portal pressure are paralleled by variations in variceal pressure [7].

Vasoactive agents primarily act by lowering of portal and variceal pressures [9]. The two major classes of drugs that are used to treat acute variceal bleeding (AVB) are vasopressin (V) and its synthetic version, terlipressin (T), and somatostatin (S) and its synthetic version octreotide (O) [7]. Terlipressin has the same properties as vasopressin, which is a strong systemic vascular constrictor that affects splanchnic circulation. However, terlipressin has a longer duration of action and fewer side effects. Somatostatin is a peptide that strongly constricts specific splanchnic vessels and stops the release of many hormones that help with digestion. Similarly, octreotide has a longer half-life and exhibits the same somatostatin activities [7].

Randomized trials have stated that both medicines are comparable to endoscopic therapy. However, the precise mechanism of how octreotide works in portal hypertension remains to be understood. Based on its presumed ability to decrease portal pressure and splanchnic blood flow, continuous intravenous (IV) octreotide therapy has been suggested to control hemorrhage from esophageal varices in cirrhotic patients. Octreotide has shown no significant effect on the wedged hepatic venous pressure, hepatic blood flow, or hepatic venous pressure gradient. Octreotide's safety profile in EVB is favorable. After being administered intravenously, terlipressin (also known as tri-glycyl-lysine vasopressin or glypressin), a long-acting vasopressin derivative with vasoconstricting action, is gradually converted to vasopressin by tissue peptidases. Terlipressin has a splanchnic vasoconstricting action that lowers portal hypertension. Unlike vasopressin, terlipressin does not activate plasminogen, reducing the risk of coronary ischemia [11]. According to hemodynamic research, terlipressin significantly and persistently lowers portal pressure and portal-collateral blood flow [13]. Intravenous injections of terlipressin can be given every four hours due to its sustained biological activity. Randomized controlled trials have demonstrated that terlipressin is as effective as balloon tamponade with a reduced incidence of side effects and is more effective than placebo, vasopressin, or vasopressin with nitroglycerin in controlling variceal bleeding. More significantly, placebo-controlled trials have demonstrated that terlipressin is the only medication that lowers mortality from variceal hemorrhage [13]. Somatostatin was introduced for the treatment of acute variceal hemorrhage because of its ability to decrease portal pressure without significant effects on the systemic hemodynamics. Numerous randomized clinical trials have demonstrated that somatostatin may be as effective as endoscopic sclerotherapy and vasopressin balloon tamponade with minimal notable adverse effects [14]. However, reports of both significant benefits and no impact have emerged from placebo-controlled trials. The variability may be due to the differences in dosage and timing of somatostatin treatment. It is well known that somatostatin boluses can result in sharp but temporary drops in gastroesophageal collateral blood flow and portal pressure; however, these effects become much less noticeable with prolonged infusions. Therefore, researchers propose that repeatedly administering somatostatin boluses at the beginning of treatment may help more people achieve controlled bleeding [15-17].

Only a few comparative studies have compared terlipressin with somatostatin thus far. Endoscopic variceal band ligation (EVL) is one endoscopic procedure performed for EVB; if EVL is unsuccessful, endoscopic injection sclerotherapy (EIS) is another alternative. Along with EVL, adjuvant pharmaceutical treatment is also considered the gold standard. EVL treatment is more effective when combined with terlipressin than EVL alone. In addition to improving treatment effectiveness, terlipressin with EVL lowers the risk of rebleeding from esophageal varices [16-29]. Considering various variables like etiology, sex, and age, the findings of the Global Burden of Disease, Injury, and Risk Factors (GBD) study on the burden of cirrhosis and its trends from 1990 to 2017 for 195 countries (GBD 2017 Cirrhosis Collaborators, 2017) showed that over 1.32 million deaths worldwide were attributable to cirrhosis, or roughly 2.4% of all deaths [17]. The results of earlier systematic reviews (SRs) and randomized clinical trials (RCTs) on mortality, bleeding control, and rebleeding concerning these vasoactive drugs remain variable. The advantages and disadvantages of vasoactive agents are not clearly defined.

Regarding efficacy, prior systematic reviews have not addressed the likelihood of bias in primary research, the application of standardized terminology, or addressing all important outcomes. Furthermore, prior systematic reviews have provided inadequate descriptions, raising concerns about safe interpretation. Lastly, several clinical guidelines advise using vasoactive drugs, but they do not specify which agent is best [7]. Even though liver transplantation is recognized to be the only effective treatment for portal hypertension, this research illustrates the importance of therapeutics for this condition and the availability of a pharmacological strategy (such as nonselective blockers, nitrates, and vasopressors). This systematic review, which evaluates the pharmacological treatments available from 1990 until 2025, was driven by the need to increase our understanding of the clinical therapeutic management of portal hypertension and variceal bleeding as its primary consequence, as demonstrated by its clinical importance. The review will allow for the mapping and compilation of current treatments, identifying potential knowledge gaps, and providing a theoretical framework for the following studies and clinical applications.

## Review

Methods

This comprehensive systematic review aims to examine and evaluate the comparative assessment between terlipressin and octreotide with respect to safety and overall clinical effectiveness in patients experiencing variceal hemorrhage with the objective of preventing adverse clinical outcomes, including rebleeding, extended hospitalization duration, and patient mortality. This systematic review was performed in compliance with the 2020 standards of the Preferred Reporting Items for Systematic Reviews and Meta-Analyses (PRISMA). 

Search Strategy

A comprehensive literature search was conducted to identify publications spanning a 35-year period (1990-2025). Databases searched included PubMed, PubMed Central (PMC), ScienceDirect Library, and Google Scholar. The search targeted articles covering randomized controlled trials (RCTs), systematic reviews, narrative reviews, cohort studies, meta-analyses, and observational studies. The terms "terlipressin", "octreotide", and "variceal bleeding" were used to formulate the search, which was then integrated using the Boolean operators "AND" and "OR." The search was refined using Medical Subject Headings (MeSH) terminology. The databases explored for article collections and the search method employed are summarized in Table 1.

**Table 1:** Databases used for collecting articles, including search strategies and appropriate filters. PMC - PubMed Central

Search Strategy	Database used	Filters used	Number of papers identified
((Terlipressin) AND (Octeriotide)) AND (Variceal Bleeding)	PMC	Full-text Meta-Analysis Observational Study Randomized Controlled Trial Systematic Review 35 years Humans	95
Terlipressin"[MeSH Terms] OR "Glipressin" OR "Gly-Gly-Gly-8-Lys-vasopressin" OR "Glycylpressin" OR "Glypressin" OR "N-(alpha)-glycyl-glycyl-glycyl-8-lysine vasopressin" OR "Remestyp" OR "TGLVP" OR "Terlipressin" OR "Terlypressin" OR "Triglycyl Lysine Vasopressin" OR "Triglycyl-(8-lysine)vasopressin" OR "Triglycylvasopressin" OR "Vasopressin, tri-Gly-8-Lys-")AND ("Octreotide"[MeSH Terms] OR "Compound 201-995" OR "Octreotide" OR "Octreotide Acetate" OR "Octreotide Acetate Salt" OR "SAN 201-995" OR "SM 201-995" OR "SMS 201-995" OR "Sandostatin" OR "Sandostatine" OR "Sandoz 201-995") AND ("Peptides, Cyclic"[MeSH Terms] OR "Circular Peptide" OR "Circular Peptides" OR "Cyclic Peptide" OR "Cyclic Peptides" OR "Cyclopeptide" OR "Cyclopeptides" OR "Orbitide" OR "Orbitides" OR "Peptides, Cyclic")AND("Esophageal and Gastric Varices"[MeSH Terms] OR "Esophageal Varices" OR "Esophageal Varix" OR "Esophageal and Gastric Varices" OR "Gastric Varices" OR "Gastric Varix") AND ("Humans"[MeSH Terms] OR "Homo sapiens" OR "Human" OR "Humans" OR "Man (Taxonomy)" OR "Man, Modern") AND ("Esophageal Diseases"[MeSH Terms] OR "Esophageal Diseases") AND ("Hypertension, Portal"[MeSH Terms] OR "Cruveilhier-Baumgarten Disease" OR "Cruveilhier-Baumgarten Syndrome" OR "Hypertension, Portal") AND ("Hemorrhage"[MeSH Terms] OR "Bleeding" OR "Hemorrhage")	PubMed	Full-text Meta-Analysis Observational Study Randomized Controlled Trial Systematic Review 35 years Humans	47
((Terlipressin) AND (Octreotide)) AND (Variceal Bleeding)	Google Scholar	Published in the last 35 years	154
((Terlipressin) AND (Octreotide)) AND (Variceal Bleeding)	ScienceDirect	Published in the last 35 years Review Articles Research Articles	358

Inclusion Criteria and Exclusion Criteria

The studies were selected for inclusion based on specific features of participants, interventions, and outcomes. For participants, a population with variceal bleeding symptoms that spans all ages, genders, and ethnicities. For interventions, the use of either octreotide or terlipressin in the demographic. And for outcomes, safety, efficacy, rebleeding, mortality, and hospital stay duration.

Papers authored and published in English, as well as translated works, including all age demographics; papers focusing on the use of octreotide or terlipressin, including outcomes focused on safety and efficacy in patients with variceal bleeding; cohort studies; randomized controlled trials; systematic reviews; observational studies; meta-analyses; narrative reviews; and case-control studies. Studies were excluded if they solely focused on cirrhotic individuals with gastrointestinal non-variceal hemorrhage, pregnant populations, animal studies, grey literature, irrelevant studies, and studies that do not pertain to the study issue or subject of interest. Research that examines a distinct Population, Intervention, Comparison, or Outcome (PICO) from those outlined in the review. Case reports or case series, conference abstracts, or posters lacking full-text publications. Research that fails to examine the designated intervention or comparator(s) of interest. Studies that do not disclose outcomes pertinent to the study topic or neglect to assess critical outcomes. Studies with limited or inadequate data extract the necessary outcome measures. Research exhibiting a significant risk of bias or inadequate methodological rigor, evaluated using suitable quality assessment instruments. Research characterized by little or deficient documentation of methodologies, outcomes, or statistical evaluations.

Results

**Figure 1:**
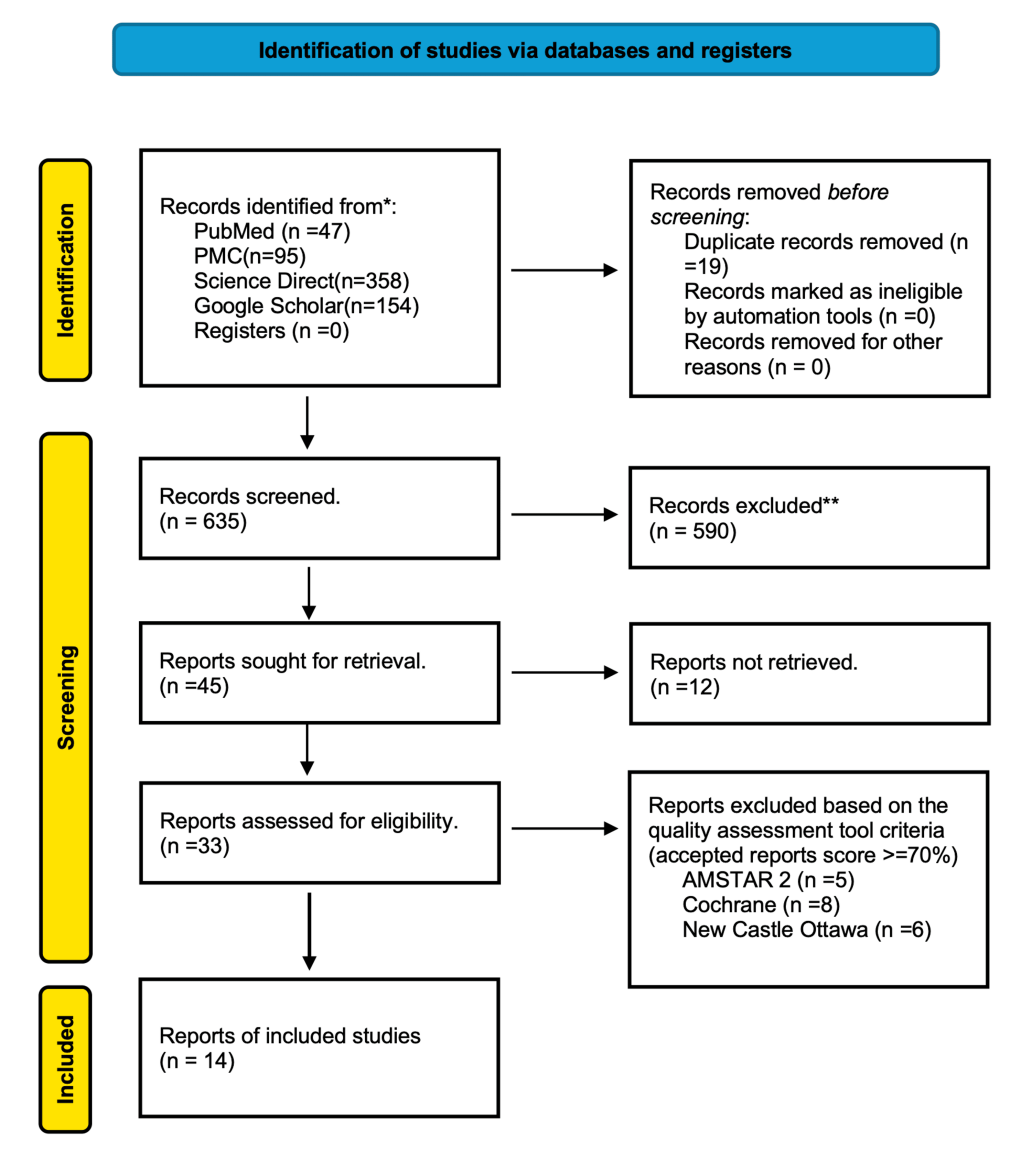
PRISMA flowchart illustrating the screening process and quality assessment of the articles PRISMA - Preferred Reporting Items for Systematic Reviews and Meta-Analyses; PMC - PubMed Central; AMSTAR 2 - A Measurement Tool to Assess Systematic Reviews 2

Employing standardized search methodologies and appropriate filters, a total of 654 articles published over the past 35 years (January 1990 to May 2025) were identified, utilizing Google Scholar's 1990-2025 filter and PubMed's filters for randomized control trials (RCTs), observational studies, meta-analyses, systematic reviews, human research, and translated and English publications. Two independent reviewers screened all records by using a pre-designed data extraction form to extract information. Study characteristics, population information, intervention/comparator descriptions, outcomes evaluated, and important findings pertinent to the review goals were among the extracted data. Disagreements among the reviewers were settled by discussion; a third reviewer was consulted for arbitration should consensus elude. The titles and abstracts of the articles were first screened for relevance based on predefined inclusion/exclusion criteria and full-text availability. After the elimination of duplicates and irrelevant records, the filtered articles underwent quality evaluation utilizing instruments such as the Cochrane Collaboration's tool for randomized controlled trials and non-randomized controlled trials, the Newcastle-Ottawa checklist for case-control and cohort studies, and the Assessment of Multiple Systematic Reviews 2 (AMSTAR 2) for systematic reviews and meta-analyses. The PRISMA flow diagram illustrating the study selection process is presented in Figure 1.

The studies included in this systematic review are summarized in Table 2. 

**Table 2:** Included studies AMSTAR - Assessment of Multiple Systematic Reviews; RCT - randomized controlled trials; SANRA - Scale for the Assessment of Narrative Review Articles; ECG - electrocardiogram; PSE - portosystemic encephalopathy; GV - gastroesophageal variceal; HCC - hepatocellular carcinoma; GVS - gastroesophageal variceal sclerotherapy; AVB - acute variceal bleeding; EGD - esophagogastroduodenoscopy; ITT - intention to treat

Author	No of participants	Study type	Study subjects with	Conclusion	Follow-up period	Quality assessment tool and score
Rehman et al. [1]	N=842	Observational study	Cirrhosis, esophageal variceal bleeding	Terlipressin and octreotide have similar outcomes in terms of control of bleeding, hospital stay, mortality, and side effects when used as adjuvant therapy for the management of variceal bleeding	6 weeks	New Castle Ottawa Scale 7/9
Huaringa-Marcelo et al. [2]	N=2431	Systematic review and meta-analysis	Cirrhosis, active variceal bleeding	In cirrhotic patients with Active Variceal Bleeding, those treated with Terlipressin-Vasopressin had a similar mortality risk compared to Octreotide-Somatostatin. However, the use of Terlipressin-Vasopressin showed an increased risk of adverse events compared to Octreotide-Somatostatin.	6 weeks	AMSTAR- 14/16
Wang et al. [3]	N=780	Systematic review and meta-analysis	Cirrhosis, esophageal variceal bleeding	There is no difference between vasopressin/ terlipressin and somatostatin/octreotide in the prevention of re-bleeding after the initial treatment of bleeding esophageal varices	5 days	AMSTAR 12/16
Dell'Era et al. [4]	N=1125	Narrative Review	Esophageal/gastric varices	Variceal bleeding is one of the most severe complications of portal hypertension correlated with liver cirrhosis, with a 6-week mortality rate of 10–20%. Primary prophylaxis is mandatory in patients with cirrhosis and high-risk esophageal varices, and when bleeding occurs, every effort should be made to arrest the hemorrhage and prevent further bleeding episodes	5 days	SANRA 11/12
Seo et al. [5]	N=780	Randomized control trial	Liver cirrhosis, active variceal bleeding	The hemostatic effects and safety did not differ significantly between terlipressin, somatostatin, and octreotide as adjuvants to endoscopic treatment in patients with acute gastroesophageal variceal bleeding.	5 days	Cochrane Low risk of bias
Sridharan et al. [6]	N=4628	Systematic review and meta-analysis	Liver cirrhosis, variceal bleeding	Terlipressin could be the best agent in the vasoconstrictor category for managing variceal bleeding. Somatostatin and vasopressin can serve as alternatives.	-----	AMSTAR 2 14/16
Nevens et al. [8]	N=not mentioned	Narrative Review	Liver cirrhosis, variceal bleeding	Vasoactive drugs should be given for 5 days. However, the beneficial effect after 48 h, if endotherapy is carried out, is not clearly shown. Due to the heterogeneity of the studies, it is not currently possible to confirm the superiority of one vasoactive compound over any of the others. The most robust evidence for therapeutic benefit is for terlipressin, including a gain in survival. However, the drug cannot be given to every patient; it should not be administered to patients whose medical history for ischemic heart disease, cerebrovascular accidents, or peripheral vascular disease is not yet clear, and where ECG monitoring is not available. Care should be taken in cases of severe hypovolemic shock.	5 days	SANRA 11/12
Wells et al. [9]	N=5,404.	Systematic review and meta-analysis	Variceal bleeding	The use of vasoactive agents was associated with a significantly lower risk of acute all-cause mortality and transfusion requirements, and improved control of bleeding and shorter hospital stay. Studies comparing different vasoactive medications failed to demonstrate a difference in efficacy.	---	AMSTAR 2 14/16
Abid et al. [12]	N=324	Randomized controlled trial	Liver cirrhosis, active variceal bleeding	The efficacy of terlipressin was not inferior to octreotide as an adjuvant therapy for the control of esophageal variceal bleed and in-hospital survival. The length of hospital stay in the terlipressin group was significantly shorter but not of any clinical importance. The predictors of prolonged hospital stay were low hemoglobin, high pulse, prolonged prothrombin time, blood at nasogastric aspirate, and PSE.	5 days	Cochrane Low risk of bias
Mahtur et al. [15]	N=1129	Systematic review and meta-analysis	Variceal bleeding	The comparison of terlipressin and octreotide showed them to be equally effective and safe therapeutic agents in patients of acute variceal bleeding. Further, evidence from future randomized controlled trials with higher quality and larger sample sizes is needed to confirm these findings.	5 days	AMSTAR 2 12/16
Hung et al. [24]	N=311	Observational	Liver cirrhosis, variceal bleeding	Terlipressin and somatostatin have similar effects on mortality when they are used as adjuvants to endoscopic treatment in cirrhotic patients with acute GV bleeding. HCC, acute renal failure, and hepatic encephalopathy are associated with elevated mortality rates after endoscopic treatment. A single session of GVS is successful for controlling bleeding in the majority of patients. Although one-fourth of patients underwent second-look endoscopy, repeated GVS may be necessary in some patients.	5 days	New Castle Ottawa Scale 7/9
Goulis et al. [27]	N=not mentioned	Narrative review	Variceal bleeding	The best evidence for an effective drug in the management of acute variceal bleeding is for terlipressin, as mortality is reduced, albeit in small trials. However, somatostatin in trials comparing it with terlipressin has a directly comparable efficacy, with fewer side effects and thus is also a drug of first choice. Furthermore, trials of somatostatin combined with sclerotherapy show that it is more effective than sclerotherapy alone and is safe, given over five days. As the therapeutic strategy of combined drug and sclerotherapy appears to have a sound basis (and there are insufficient data for terlipressin used over five days), so somatostatin combined with sclerotherapy represents the optimal therapy today.	5 days	SANRA 11/12
Gross et al. [28]	N=1334	Systematic review and meta-analysis	Variceal bleeding	Ligation is the most effective treatment option. No significant difference was found between the efficacy of sclerotherapy and treatment with somatostatin or octreotide.	5 days	AMSTAR 13/16
Zhou et al. [30]	N=3344	Systematic review and meta-analysis	Active variceal bleeding	Our findings were in accordance with the current recommendations regarding terlipressin for the treatment of AVB in cirrhosis. However, due to the low quality of evidence, further studies are recommended	42 days	AMSTAR 13/16

To ensure methodological transparency, we conducted a risk of bias and quality assessment for all included studies using tools appropriate to their design. Systematic reviews and meta-analyses were appraised using the AMSTAR 2 tool. Randomized controlled trials were evaluated according to the Cochrane Risk of Bias 2.0 framework. Observational studies were assessed using the Newcastle-Ottawa Scale (NOS), and narrative reviews were appraised using the Scale for the Assessment of Narrative Review Articles (SANRA) criteria. The results are presented in Tables 3-6. 

**Table 3:** Quality Appraisal of Systematic Reviews and Meta-Analyses Using the AMSTAR 2 Tool RCT - randomized controlled trial; AMSTAR 2 - A Measurement Tool to Assess Systematic Reviews version 2

Author(s)	Study design	AMSTAR 2 Score (out of 16)
Huaringa-Marcelo et al. [2]	Systematic review and meta-analysis (7 RCTs)	14
Wang et al. [3]	Systematic review and meta-analysis (RCTs + observational studies)	12
Sridharan et al. [6]	Network meta-analysis (included RCTs only)	14
Wells et al. [9]	Narrative review with comparative analysis	14
Mathur et al. [15]	Systematic review (mixed interventional studies)	12
Gross et al. [28]	Systematic review (older trials and non-comparative studies)	13
Zhou et al. [30]	Systematic review and meta-analysis (pooled RCTs)	13

**Table 4:** Risk of bias assessment of included randomized controlled trials using the Cochrane RoB 2.0 tool RoB 2.0 - Risk of Bias 2.0; Low - Low risk of bias across all domains; Some Concerns - At least one domain raises some concern, but no high risk; High - At least one domain is at high risk of bias, or multiple domains raise some concern

Study	Randomization process	Deviations from intended interventions	Missing outcome data	Measurement of outcome	Selection of reported result	Overall risk of bias
Seo et al. [5]	Low	Low	Low	Low	Low	Low
Abid et al. [12]	Low	Low	Low	Low	Low	Low

**Table 5:** Quality assessment of observational studies using the Newcastle-Ottawa Scale (NOS) NOS - Newcastle-Ottawa Scale, a tool for assessing the quality of non-randomized studies in meta-analyses

Study	Selection (max 4)	Comparability (max 2)	Outcome (max 3)	Total Score (out of 9)	Quality
Rheman et al. [1]	3	2	2	7	High
Hung et al. [24]	3	2	2	7	High

**Table 6:** Quality assessment of narrative reviews using the Scale for the Assessment of Narrative Review Articles (SANRA) tool SANRA - Scale for the Assessment of Narrative Review Articles

Study	Justification of importance (0–2)	Statement of aims (0–2)	Literature search description (0–2)	Referencing quality (0–2)	Scientific reasoning (0–2)	Data presentation (0–2)	Total score (out of 12)	Quality
Dell'Era et al. [4]	2	2	1	2	2	2	11	High
Nevens et al. [8]	2	2	1	2	2	2	11	High
Goulis et al. [27]	2	2	1	2	2	2	11	High

Discussion

Pathophysiology of Variceal Bleeding

Portal hypertension (PH) leads to a decline of liver function, clinically manifesting as symptoms such as ascites, jaundice, hepatic encephalopathy, hepato-renal syndrome, and gastrointestinal bleeding. Prolonged hepatic damage, increased portal pressure, and a high degree of portosystemic shunting are the main causes of the development of varices. Varices start as tiny formations and progressively grow larger over time until they reach a noticeable size; at this point, they usually burst and lead to bleeding. Variceal bleeding is a frequent cause of other cirrhosis-related problems, such as hepatorenal syndrome and bacterial infections; it frequently causes a decline of liver function. Key predictive factors for the onset of variceal bleeding include the size of the varices, the degree of hepatic dysfunction (as indicated by the Child-Pugh score), hepatic venous pressure gradient (HVPG) exceeding 16 mmHg, coagulopathy, and the severity of the underlying condition [18,19]. The North Italian Endoscopic Club (NIEC) index integrates these risk indicators, facilitating the classification of patients into various groups based on their predicted one-year bleeding risk, which spans from six to 76%. In summary, the size of varices is the most significant predictor of variceal bleeding [18]. Generally, esophageal varices experience bleeding more often than gastric varices; however, when gastric varices do bleed, the events are typically more severe.

**Figure 2:**
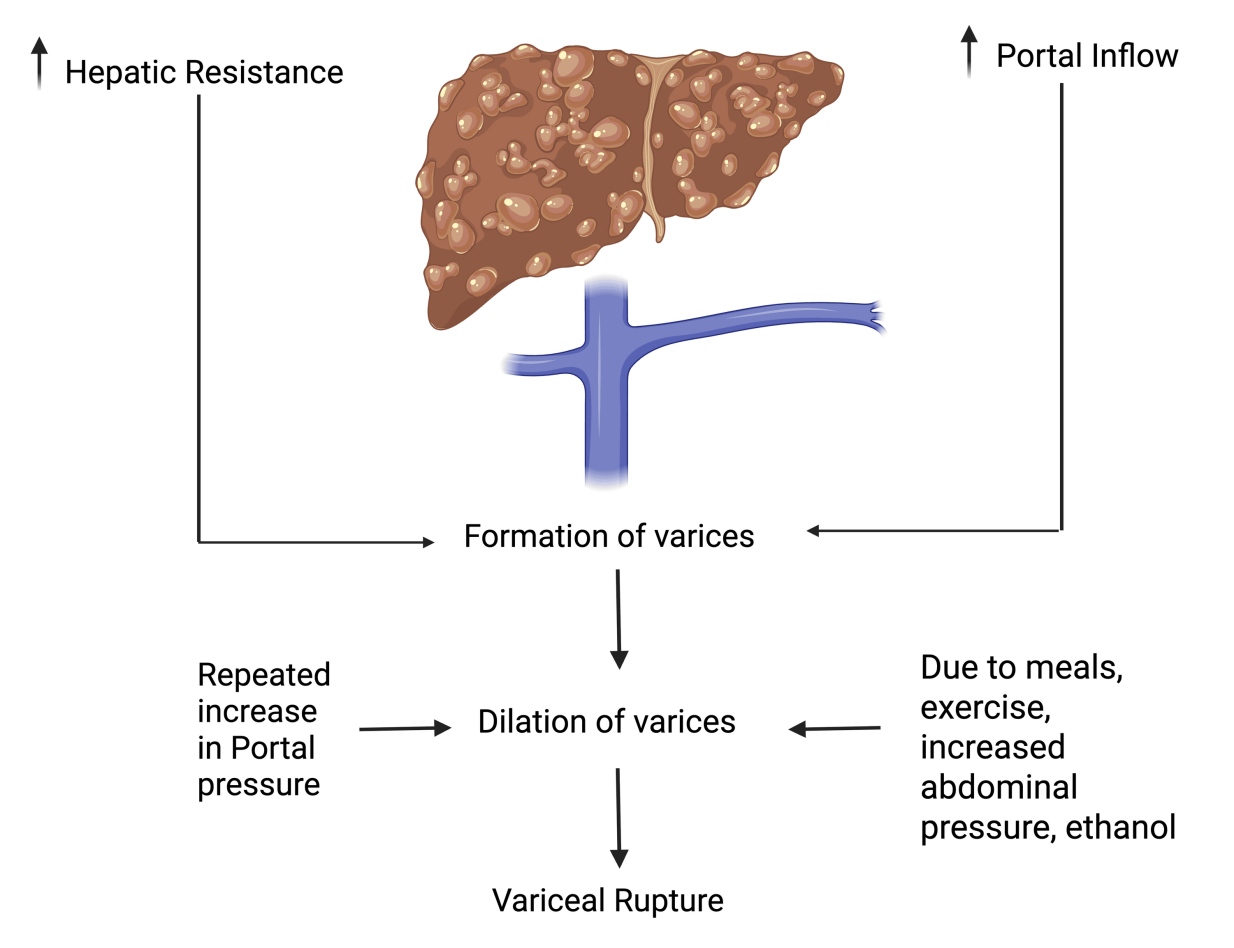
Pathophysiology of variceal bleeding Created with BioRender.com by author Shivling Swami

The primary objectives for patients experiencing active bleeding are establishing control over the hemorrhage, ensuring hemodynamic stability, and averting complications from blood loss. As previously noted, variceal bleeding infrequently occurs when the HVPG stays under 12 mmHg, with 16 mmHg identified as a risk factor for the onset of bleeding [19]. The pathophysiology of variceal bleeding is summarized in Figure 2.

Mechanism of Action of Vasoactive Agents

The primary vasoactive agents used in the control of acute variceal bleeding include vasopressin, somatostatin, terlipressin, and octreotide. Terlipressin, a synthetic vasopressin analogue, functions as a potent vasoconstrictor that reduces portal blood flow and induces splanchnic vasoconstriction, which lowers portal pressure. Terlipressin's side effects, which affect almost 25% of patients, include abdominal cramps, diarrhea, bradycardia, and hypertension, which are among the generally mild side effects. About 2-4% of cases, however, result in more serious side effects that require cessation of the medication, such as arrhythmias, angina, and limb ischemia. An electrocardiogram (ECG) must be performed before starting terlipressin, and patients classified as high-risk must have cardiac monitoring. According to current recommendations, terlipressin should be started at 2 mg every four to six hours for the first 48 hours. To reduce the risk of premature rebleeding, the treatment may be continued for a maximum of five days at a dosage of 1 mg every four to six hours. Portal pressure is successfully reduced by terlipressin, and this effect is still noticeable four hours after administration. According to clinical studies, even without endoscopic intervention, 75-80% of cases of acute variceal bleeding are successfully managed within 48 hours, and 67% are successfully managed by day five. Compared to a placebo or the lack of an active medication, terlipressin improves six-week survival rates and bleeding control; according to a meta-analysis and several studies, it is a safer option than vasopressin and nitroglycerin. Octreotide is a somatostatin analogue and can lower portal pressure and collateral blood flow by blocking the release of splanchnic vasodilatory peptides (like glucagon) and boosting the actions of endogenous vasoconstrictor systems. Additionally, octreotide prevents the increase in portal blood flow and portal pressure after a meal. Fifty µg of octreotide should be given as an intravenous bolus first and then continuously at 50 µg per hour. Octreotide shows no discernible effect on mortality rates. However, it effectively prevents early rebleeding. It is hypothesized that octreotide's beneficial effects may be related to its capacity to reduce the postprandial increase in portal pressure. According to clinical trials, octreotide is ineffective when used without endoscopic treatment. Four randomized controlled trials found that optical coherence tomography may improve the efficacy of endoscopic therapy by lowering the risk of early rebleeding. However, it appears to have minimal or no impact when used as a standalone treatment, particularly as an initial intervention, likely due to tachyphylaxis.

**Figure 3:**
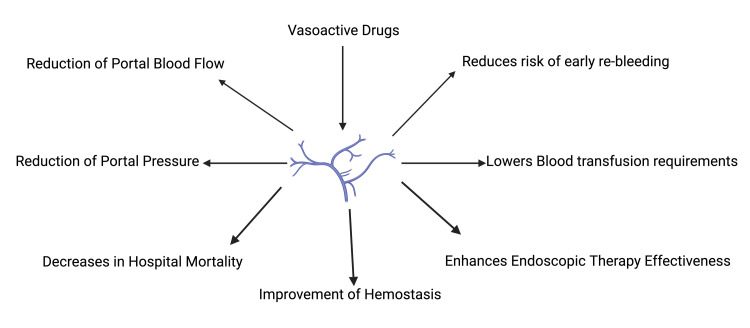
Summary of the therapeutic effects of vasoactive drugs in variceal bleeding Created with BioRender.com by author Shivling Swami

In two randomized controlled trials, octreotide demonstrated superior efficacy to vasopressin while showing comparable results to terlipressin in managing bleeding control. Side effects associated with octreotide were less frequent and less severe [5,9,11,12,18]. In summary, vasoactive drugs reduce rebleeding, lower blood transfusion requirements, enhance endoscopic therapy effectiveness, improve hemostasis, decrease in-hospital mortality, reduce portal pressure, and reduce portal blood flow [5-30]. The following beneficial effects and roles of vasoactive drugs have been summarized below in Figure 3.

Analysis

Overview of Vasoactive Agents in Acute Variceal Bleeding: Efficacy and Safety Profiles

Many studies have been carried out to directly compare the effectiveness of octreotide (O) and terlipressin (T). Terlipressin markedly decreased variceal pressure compared to octreotide in a series of trials, so it is suggested as the primary option, while octreotide or somatostatin is advised as the secondary option [5-7,16]. Nonetheless, no substantial differences in treatment responses were seen among patients with variceal hemorrhage in several additional investigations evaluating distinct vasoactive medications used as monotherapy [1,3,9,10]. There is strong evidence that the risk of all-cause mortality in cirrhotic patients with acute variceal bleeding (AVB) is similar for both vasoactive agent groups (terlipressin-vasopressin vs. octreotide-somatostatin). A meta-analysis by Wang et al. [3] reported a pooled relative risk of 0.96 (95% CI: 0.81-1.14; p=0.64), indicating no statistically significant difference in mortality outcomes between the two groups [3]. Nearly all primary studies showed a higher incidence of adverse events in the terlipressin-vasopressin (T-V) group, with the majority showing statistically significant outcomes despite observing moderate statistical heterogeneity. Given its intense systemic vasoconstrictive action, the observed results could be related to the administration technique and its ischemic effects [3]. This result was also noted in another study as well [1]. Although terlipressin is known for its potent vasoconstrictive effects, it can also cause serious dysrhythmias and ischemic complications as a result it cannot be given to every patient; it should not be given to those with an unclear medical history concerning ischemic heart disease, cerebrovascular accidents, or peripheral vascular disease, especially in the lack of ECG monitoring. In cases of extreme hypovolemic shock, one should use great care [1,9]. Terlipressin had more complications than somatostatin, but severe adverse events were not more common. Most terlipressin side effects were mild and reversible. The choice between octreotide and somatostatin depends on the drug's availability. Terlipressin use has a more frequent occurrence of hyponatremia, which matches results from previous studies [6]. Terlipressin is still a beneficial choice if side effects are carefully considered. On a similar thought, vasopressin can still be used, but it must be closely monitored for adverse outcomes. Terlipressin-Vasopressin could be a good choice for cirrhotic patients with AVB and related side effects like hepatorenal syndrome or hypotension. Terlipressin has a lower risk of complications than vasopressin because of its gradual drug release and less noticeable effect on systemic hemodynamics. Compared to vasopressin and terlipressin, it was found that somatostatin and octreotide were linked to a lower incidence of non-serious adverse events. However, more research is needed to support this assertion [3,7,9,30].

Prognostic Indicators and Secondary Outcomes in Acute Variceal Bleeding

In the present group of variceal hemorrhage patients, octreotide was associated with a slightly more extended hospital stay than terlipressin. The study conducted by Rehman et al. analyzed individual factors that could increase hospital stay duration. Multivariate Cox regression modeling showed that there were many factors that led to longer hospital stays, such as a faster heart rate, higher bilirubin levels, cardiovascular problems during admission, and the appearance of portosystemic encephalopathy (PSE) upon arrival. Emergency department records of tachycardia showed a link to longer stays in the hospital. Also, there were links between long-term institutional care and both hyperbilirubinemia and PSE at the first assessment. These identified variables are signs of more advanced disease severity, which could affect the timeframes for therapeutic management. Cardiovascular events during admission, the occurrence of PSE, the successful stopping of variceal hemorrhage after an Esophagogastroduodenoscopy (EGD), total bilirubin levels, and serum albumin levels all turned out to be independent predictors of six-week survival outcomes. Hypoalbuminemia was linked to a higher risk of death. For this reason, when treating esophageal variceal bleeding grade 2 (EBV2), albumin is used in combination with terlipressin or octreotide. They also found that a shorter hospital stay was associated with controlling variceal hemorrhage after EGD [1]. The secondary outcomes for blood transfusion, early and late rebleeding, bleeding control, and length of hospital stay showed no significant differences, and the certainty of the evidence for these outcomes was rated as low or very low [1,3].

Mortality Outcomes and Bleeding Control Across Vasoactive Agents

In the 2019 network meta-analysis by Sridharan et al. [6], which included more than 50 randomized control trials, found that mixed treatment pooled estimates revealed no appreciable differences in the mortality risk between any of the vasoactive drugs and a placebo. However, the direct pooled estimates revealed that terlipressin was linked to a reduced mortality risk (OR: 0.61; 95% CI: 0.43-0.87; p<0.01), while somatostatin also showed mortality benefit (OR: 0.65; 95% CI: 0.46-0.91; p<0.05). Moreover, the subgroup study of mortality risks at 24 hours revealed a significant drop in terlipressin and somatostatin. The likelihood of mortality was not much changed by vasoactive administration times before or after endoscopic procedures [6]. In 2014, Seo et al. compared terlipressin, octreotide, and somatostatin simultaneously in one study; they found that many past randomized studies and meta-analyses show that among those with variceal bleeding, terlipressin is the only drug that provides a survival benefit above placebo. This has prompted some authors to advocate terlipressin as the leading choice and suggest somatostatin or octreotide as the secondary one. Nevertheless, many more studies assessing the clinical efficacy of different vasoactive drugs used as monotherapy have found no variations in mortality rates [6,28,29]. When used in conjunction with endoscopic therapy, the mortality rate stays statistically similar between terlipressin and somatostatin or octreotide. Consequently, any of the three splanchnic vasoconstrictors, terlipressin, somatostatin, and octreotide, may be used alternately as an adjunctive treatment alongside conventional endoscopic therapies for managing gastroesophageal variceal hemorrhage. According to previous systematic reviews, patients who received endoscopic therapy along with a vasoactive agent were more likely to achieve bleeding control than those who received treatment with a vasoactive agent alone. Patients who received octreotide-somatostatin (O-S) regimens were more likely to achieve bleeding control than those receiving terlipressin-vasopressin (T-V) combinations [3]. However, a network meta-analysis done by Huaringa-Marcelo et al. [2], which included seven randomized controlled trials, found that terlipressin was significantly more effective than octreotide in achieving initial bleeding control (OR: 1.44; 95% CI: 1.02-2.03; p<0.05). No significant differences were observed in rebleeding rates (OR: 1.21; 95% CI: 0.88-1.66) or all-cause mortality. Adverse events such as abdominal pain and ischemic complications were more frequently associated with terlipressin. These findings would suggest that even though terlipressin offers short-term benefit, its use should be considered with caution in patients with underlying cardiovascular comorbidities due to its side effect profile. In a double-blinded randomized control trial conducted by Feu et al. in 1996, they demonstrated that somatostatin and terlipressin successfully reduce acute variceal hemorrhage while preserving a low mortality rate [26]. This is further supported by the results obtained in a systematic review, which showed that vasoactive agents are linked to a lower risk of all-cause death and transfusion needs, improved bleeding control, and a shortened hospital stay [10]. In a narrative review performed by Nevens et al., they concluded that when suspicion of variceal bleeding strikes, it is imperative to start vasoactive drugs routinely and right away [24]. *Efficacy of Vasoactive Agents in Rebleeding Control*

A 2015 meta-analysis compared terlipressin-vasopressin (T-V) against octreotide-somatostatin (O-S) in preventing variceal rebleeding at five days before and five days following the management of the first bleeding incident. The results demonstrated that, in preventing rebleeding both inside and outside five days after the first control of bleeding, terlipressin-vasopressin had no appreciable effect over octreotide-somatostatin [4]. This study aimed to reduce variability by focusing on the treatment of rebleeding at two specific intervals: five days before and five days after the intervention. Research indicates that endoscopic variceal ligation is more effective than endoscopic sclerotherapy in managing esophageal variceal hemorrhage. Timely endoscopy coupled with banding is expected to reduce rebleeding rates compared to other treatments. Three studies gave a clear account of the endoscopic treatment used, and the endoscopic techniques differed among the studies. In preventing rebleeding following the first treatment of bleeding esophageal varices, this meta-analysis found no difference between vasopressin/terlipressin and somatostatin/octreotide. This study has considerable restrictions, including possible bias, a small sample size, and a lack of endoscopic treatment criteria. Furthermore, the absence of sensitivity analysis, sequence generation of randomization, and intention-to-treat (ITT) analysis in any study casts doubt on the validity of the results. Their study was limited in using heterogeneous time points to evaluate various outcomes across the included studies, possibly distorting the results [10]. Improved hemostasis was associated with somatostatin and vasopressin, while terlipressin and vasopressin significantly reduced the need for blood transfusions. Furthermore, terlipressin greatly reduced the rebleeding rate [7]. In a narrative review conducted by Dell'Era et al., they concluded that the choice of vasoactive drugs should be based on local resources at their disposal. Antibiotic prophylaxis is crucial since many variceal bleeders show a bacterial infection upon admission or develop one during the following week; even in the absence of infection, antibiotics should be given [5,9]. Medications of choice are cephalosporins. As beta-blockers help to lower portal pressure, they are a pillar of medical therapy in the framework of primary and secondary prophylaxis. Some patients show contraindications to these drugs, and occasionally, adverse events occur, which leads to the therapy needing to be stopped. Band ligation, beta-blockers, or both are recommended to prevent recurrent bleeding. In this regard, assessing the hemodynamic response to drug treatment provides prognostic information about the probability of rebleeding. Because they effectively lower portal pressure [5]. Conducting diagnostic endoscopy helps one confirm the diagnosis and assess the degree of bleeding. If possible, endotherapy such as sclerotherapy or ligation must be done in cases of active variceal bleeding seen during endoscopy. In the absence of active bleeding symptoms, the clarity regarding the beneficial effect of endo-therapy and medical treatment remains doubtful. Drugs classified as vasoactive should be taken over five days. Still, the beneficial effect after 48 hours of endo-therapy is unclear [9]. *Endoscopic Band Ligation and Adjunctive Vasoactive Therapy: Survival Impact*

In a double-blinded, randomized, placebo-controlled trial carried out in 2009, the patients undergoing esophageal variceal bleeding (EVB) who combined terlipressin or octreotide with endoscopic band ligation proved effective at 94.13%. As a supplementary treatment for controlling EVB and guaranteeing in-hospital survival, terlipressin proved to be as effective as octreotide. Although this difference lacked clinical significance, the duration of hospitalization in the terlipressin cohort was reduced. Decreased hemoglobin levels, elevated pulse rates, longer prothrombin time, presence of blood in nasogastric aspirate, and PSE were associated with prolonged hospital stays [13]. Future high-quality research is required to ascertain the efficiency of medical therapies for avoiding rebleeding from esophageal and gastric varices [4,13]. *Somatostatin Dosing in Emergency Endoscopy: Efficacy Considerations*

Greater dosages of somatostatin infusion (500 µg/h) produced a higher rate of hemostasis and a lower risk of mortality, according to a previous study on cirrhotic patients with active variceal hemorrhage during emergency endoscopy. Seo et al. concluded that more research is needed to verify the optimal dosage of somatostatin in patients with acute variceal bleeding, even though in the present study, they obtained similar results with a lower dosage of somatostatin compared to other vasoactive drugs [26]. *Optimizing Acute Management: Terlipressin, Octreotide, and Endoscopic Strategies*

A 2021 study involving a total of 2431 patients found no considerable difference between terlipressin and octreotide in post-endoscopy bleeding control outcomes. According to the study, both drugs may be used as the mainstay of care for acute variceal hemorrhage. Endoscopic injection therapy might be postponed for numerous individuals or employed exclusively when pharmacological interventions fail to manage variceal bleeding episodes. Moreover, the minimal occurrence of severe complications indicates that either medication could be administered over extended durations to avoid early variceal hemorrhage recurrence [7]. The use of somatostatin or octreotide in patients suspected of variceal hemorrhage could be recommended as a primary treatment option when ligation is not easily accessible, given the advantages of medical intervention during emergencies, such as immediate accessibility, no specialized knowledge necessary, and no requirement for specific tools. If the diagnosis is accurate, the likelihood of stopping the bleeding is at least 68%. Controlling the bleeding dramatically improves the conditions for the subsequent endoscopy. If the diagnosis of AVH is later found to be incorrect (e.g., peptic ulcer disease), administration of these drugs still poses minimal risk. However, this algorithm needs to be evaluated through prospective randomized studies. Even if medical treatment is successful, a follow-up endoscopy should be performed as soon as possible to rule out other bleeding sources and, if the diagnosis is correct, to perform endoscopic therapy, preferably ligation. The necessity for endoscopic therapy stems from the long-term recurrence rate of variceal bleeding when the varices remain untreated [24]. A 2018 meta-analysis done by Zhou et al. [30] compared the efficacy of terlipressin and octreotide in achieving bleeding control at 24 hours in patients with acute variceal bleeding. The study found that terlipressin was significantly less successful in controlling bleeding than octreotide (95% CI: 0.48-0.94; p=0.021. However, it should be noted that this meta-analysis only included two studies on the control of bleeding within 24 hours, published more than 20 years ago, and involved a relatively small patient population, so this finding needs to be interpreted cautiously. The combination of terlipressin and endoscopic variceal ligation (EVL) reduced the need for transfusions and five-day treatment failure (OR=14.46, p=0.01). In contrast, there was no significant difference between the sclerotherapy and terlipressin groups. Octreotide was as effective as sclerotherapy in treating AVB and led to fewer complications. Terlipressin is more effective than balloon tamponade at reducing rebleeding (OR=0.05, p=0.001) and the need for transfusions with a statistically significant weighted mean difference (WMD=-2.70, p=0.02). However, if terlipressin is ineffective, balloon tamponade should be considered a short-term solution. Additionally, balloon tamponade may negatively affect the survival rates of patients with AVB and cause discomfort for patients [30].

In conclusion, we would like to say that in treating AVB in cirrhotic patients, this systematic review has compared the efficacy and safety profiles of terlipressin, octreotide, somatostatin, and vasopressin. Although some research pointed towards a better decrease in variceal pressure and possible survival advantage with terlipressin, most of the data shows no significant variations in general mortality, rebleeding rates, bleeding control, or hospital stay length between the various vasoactive agents when used as part of combination therapy with endoscopic intervention. Importantly, terlipressin was linked to a greater frequency of adverse events, which were mostly mild and reversible, than somatostatin and octreotide, whose side effect profiles were relatively more favorable. The decision between terlipressin-vasopressin and octreotide-somatostatin should be based on patient comorbidities, medication availability, and resource settings, since both remain successful first-line treatments for AVB. Patients with cardiovascular or ischemic risk factors should exercise care while using terlipressin. To optimize hemostatic control and maximize clinical outcomes, early administration of vasoactive agents in suspected instances is essential, even prior to endoscopy.

Moreover, while terlipressin helped to lower in-hospital death and control bleeding within 48 hours, stronger and modern randomized controlled studies are required to confirm its superiority over other drugs. Future high-quality research is justified to clarify the best vasoactive agent choice, dosing policies, and cost-effectiveness, especially in different clinical settings, given the slight variations in primary clinical outcomes and the low confidence of evidence for several secondary outcomes.

Ultimately, terlipressin and octreotide, the key vasoactive drugs, are vital for the adjunctive management of AVB. Therapy should be based on clinical judgment, patient-specific considerations, and local availability until more evidence appears. Therefore, early intervention and combining pharmacologic therapy with timely endoscopic treatment to maximize patient outcomes should be guaranteed.

Limitations and strengths

The conclusions of this systematic review cannot be considered without a few limitations. The quality of the evidence differed significantly. Several included studies had small sample sizes, possible biases from lack of blinding, and incomplete reporting of randomization techniques or intention-to-treat analyses. Furthermore, many studies fell short in this regard due to a lack of thorough reporting on patient comorbidities, especially cardiovascular risk factors, which are vital when assessing drugs like terlipressin with recognized systemic vasoconstrictive effects. Differences in adjunctive therapies might have affected patient outcomes, including antibiotic prophylaxis, beta-blocker use, and endoscopic intervention timing, which were not consistently controlled across studies. The studies ranging from 1990 to 2025 were included in this paper, with routine standards of care varying from one study to another and from one time interval to another, and numerous trials assessed were conducted over an extended duration; certain earlier studies may have exhibited inconsistent medical care regarding the utilization of diagnostic and/or therapeutic endoscopy, balloon tamponade, and antibiotic prophylaxis for spontaneous bacterial peritonitis, restricting the direct relevance of certain results to present clinical practice. Finally, while a network meta-analysis offered insightful comparisons, trial sequential analyses indicated that more high-quality randomized controlled trials are still needed to verify several important conclusions, particularly on mortality advantages, optimal dosing strategies, and long-term consequences, including rebleeding rates and hospital stay lengths.

Despite the above limitations, this systematic analysis has certain benefits. It is one of the most comprehensive syntheses of the present data comparing vasoactive medications in the management of acute variceal bleeding. By including data from multiple high-quality systematic reviews, meta-analyses, and randomized controlled trials, this paper provides a strong overview of the comparative efficacy and safety profiles of terlipressin and octreotide. The review methodically evaluated bleeding control and clinically significant outcomes, including mortality, adverse events, and the need for rescue therapies, thereby offering a more complete knowledge of the therapeutic environment. Efforts were made to critically assess the methodological quality of the included studies, ensuring that findings were grasped considering study design strengths and weaknesses. Wherever feasible, trial sequential analysis findings were highlighted to provide insight into the strength and conclusiveness of meta-analytic findings. By discussing the effect of adjunctive therapies, the timing of intervention, and evolving standards of care, the review helps to frame its results in modern clinical practice; therefore, enhancing their relevance for current decision-making. This study emphasizes certain knowledge gaps in the literature and areas of ongoing uncertainty, so guiding future research and helping to shape the next randomized controlled trials in this important clinical domain.

## Conclusions

This systematic review underscores the fact that both octreotide and terlipressin are effective vasoactive agents when they are used as adjuncts to endoscopic therapy in the management of acute variceal bleeding (AVB) among cirrhotic patients. While some studies suggest that terlipressin may offer positive hemodynamic advantages, including faster bleeding control and a potential mortality benefit, the overall evidence from studies does not demonstrate consistent superiority concerning rebleeding rates, mortality, or hospital stay duration compared to octreotide. In some studies, terlipressin is associated with a higher incidence of adverse events; however, these are mild and reversible. Patients with cardiovascular risk factors should exercise caution when using terlipressin. Given the comparable efficacy and safety profiles, the choice between terlipressin and octreotide should be based on the clinical context, drug availability, and individual patient risk factors. Early initiation of pharmacologic therapy, ideally before endoscopy, remains a critical aspect for optimizing positive outcomes. Despite existing data, several limitations exist in study quality and heterogeneity, which make it necessary to conduct further high-quality, large-scale, randomized controlled trials. Future studies should clarify optimal dosing strategies, long-term outcomes, and cost-effectiveness across various clinical settings. Until then, individualized, timely therapy using either agent remains the cornerstone of managing acute variceal hemorrhage.
